# Breastfeeding and Its Influence on Psychomotor Development: An Investigation Based on the LAyDI Study (PAPenRed)

**DOI:** 10.3390/nu17060967

**Published:** 2025-03-10

**Authors:** Silvia Martín-Ramos, Begoña Domínguez-Aurrecoechea, Marta Carballal-Mariño, Guadalupe Del Castillo-Aguas, Gonzalo Solís-Sánchez, on behalf of the “Red de Investigación en Pediatría de Atención Primaria” (PAPenRed)

**Affiliations:** 1 Neonatology Department, Hospital Río Hortega, 47012 Valladolid, Spain; silvia_1651991@hotmail.com; 2 PAPenRed Coordinating Team, Institute for Health Research of the Principality of Asturias (ISPA), 33011 Oviedo, Spain; begoa.dominguez@gmail.com; 3 Novo Mesoeiro Health Centre, 15190 A Coruña, Spain; mcarballalmarino@gmail.com; 4 Carihuela Health Center, 29620 Torremolinos, Spain; gdelcastilloaguas@gmail.com; 5 Central University Hospital of Asturias, Institute for Health Research of the Principality of Asturias (ISPA), RICORS Net, University of Oviedo, 33003 Oviedo, Spain

**Keywords:** breastfeeding, nutrition, psychomotor development, infants, young toddlers

## Abstract

**Objectives:** To analyse whether breastfeeding (BF) is related to better psychomotor development in the first two years of life. **Methods:** Prospective longitudinal study of a cohort of children born in Spain (between April 2017 and March 2018) and followed during the first two years of life by their primary care paediatrician in eight visits (LAyDI study—PAPenRed research network). The Haizea-Llevant development chart was used to assess the psychomotor development (DPM), and the subjects were divided according to whether or not they met each milestone. **Results:** The initial sample was 1946 children (50.1% boys), which varied at each visit from 1946 on the first and second visit to 1076 on the last visit; DPM at 12, 18 and 24 months was compared according to the type of BF at 6 months; at 24 months, significant differences were found in the achievement of milestones in the BF-at-6-months group (“scribbles spontaneously”, *p* 0.007 and “descends stair”, *p* 0.002). When comparing the mean duration of BF and exclusive breastfeeding, according to milestones reached or not, statistically significant differences were observed in more milestones at the 24-month visit, including “eats with a spoon” (5.6 months in the group that reached it vs. 2.4 months in the group that did not, *p* 0.014), and again for the milestone ‘scribbles spontaneously’ (5.6 months vs. 1.8 months, *p* 0.021), among others. **Conclusions:** In our study, psychomotor development in the first two years of life does not show major differences in relation to the type of feeding; from this age onwards, the differences may be greater. Many factors influence psychomotor development in the first years of life, with breastfeeding not being an isolated factor.

## 1. Introduction

The numerous benefits of breastfeeding (BF) are widely known, both for infants’ physical health and for their cognitive and motor development; much work is currently being undertaken to try to promote breastfeeding in the health sector because of its known advantages [1]. Some studies show that breastfed infants perform better on tests of psychomotor development during infancy and cognitive development later in life [2,3]. Breast milk contains essential components for neuronal function (docosahexaenoic acid, DHA, among others) that are essential for proper brain development, and studies have shown that BF contributes, in particular, to an improvement in cerebral white matter, which contributes to improvements in motor and cognitive skills [4].

Longer duration of breastfeeding, according to some studies, is associated with better scores on assessments of fine and gross motor development during early infancy [5,6], and breastfeeding during the first months has also been associated with a lower incidence of psychomotor developmental delays and greater emotional development [7]. It should always be borne in mind that all this will depend on multiple factors, including socioeconomic and genetic factors, which is why a study conducted in Spain that evaluated more than 1000 children found that longer duration of breastfeeding is related to better scores on scales of psychomotor and cognitive development, after adjusting for socioeconomic and maternal factors [8].

Other known benefits of BF are the prevention of infections, which in turn may prevent disruptions in neurodevelopment due to severe illness in early childhood [3,9].

However, although it is clear from most studies, some authors in their studies reflect that it may be unlikely that breastfeeding is associated with higher intelligence or better psychomotor development [10,11].

Therefore, our main objective with this study is, taking advantage of a large and varied cohort of children, to investigate whether BF improves psychomotor development in the first two years of life and, in particular, whether the duration of BF and exclusive BF improve psychomotor development at follow-up.

## 2. Materials and Methods

### 2.1. Study Design

Our study is based on a prospective longitudinal study of a cohort of children born between April 2017 and March 2018 who attended primary care (PC) paediatricians in the PAPenRed research network. Known as the LAyDI study, it was created to investigate the influence of socioeconomic, cultural and individual factors on the initiation and duration of breastfeeding.

The LAyDI study [12,13] is a nationally stratified, two-stage sampling. Each paediatrician collected 12 newborns for a sample size of 1500 newborns (for a finite population of 426,303 children born in Spain in 2015, with a margin of error of 2.5% at a 95% confidence level).

### 2.2. Participants

The inclusion criteria were attendance at the PC paediatric clinic during the study period and having made the first and second visit. Excluded were preterm births of less than 37 weeks, low birth weight (less than 2400 g in boys and 2100 g in girls), multiple births, admission to maternity or neonatology for more than 5 days, malformations or serious pathology in newborns, children whose mothers had moderate to severe health problems during pregnancy or the puerperal period, and children of mothers with oral and written language limitations in Spanish.

### 2.3. Variables

The LAyDI study analysed variables related to gestation, delivery, the neonatal period, social, economic and biological variables. Data were collected in paediatricians’ consultations at the first visit (before 15 days of life) and at subsequent visits at 1, 2, 4, 6, 12, 18 and 24 months.

Of the variables collected, variables related to psychomotor development from the 4-month visit onwards were selected for this article; these variables are each of the items included in the Haizea-Llevant development chart, which assesses the level of development in the cognitive, social and motor spheres of children aged 0 to 5 years, dividing the items into the areas of socialisation, manipulation, language and logic, and postural area [14]. The data were collected by the paediatrician and the collaborating nurse, with specific indications for the assessment of each of the items; the possible answers for each of the items were as follows: not acquired, yes acquired, not assessable.

In this study, at each visit, the different psychomotor milestones were compared for the following groups: children fed with formula (FF), exclusive breastfeeding (EBF), or any type of breastfeeding (BF); BF also includes all types of breastfeeding from 6 months onwards. The groups were also compared according to the duration of BF (greater than or equal to 6 months or less); and finally, were compared depending on the means of duration of breastfeeding and exclusive breastfeeding for each developmental milestone.

### 2.4. Data Analysis

Qualitative variables were analysed with frequencies (percentages). Univariate analyses were performed using the appropriate tests for each situation: Pearson’s Chi-square test, Fisher’s exact test, and the Mann–Whitney U test. The analyses were performed with the SPSS v.21 statistical package (IBM SPSS Statistics software), and a *p*-value was considered significant when it was <0.05.

### 2.5. Ethical Considerations

The PAPenRED project obtained the approval of the Ethics and Scientific Research Committee of Aragón (Approval Report, Act No. 19/2013; C.P.-C.I. PI13/00154), and the present study has been approved by the Research Ethics Committee of the Principality of Asturias (study No. 213/16). It also received approval from the Central Research Commission of the Madrid Health Service (23 February 2017). The mother was responsible for signing the informed consent form at the first visit. Each child was assigned a unique code for correct anonymisation.

## 3. Results

The initial sample analysed was 1946 infants (50.1% male), with a mean gestational age of 39.3 weeks. Table 1 shows the evolution of breastfeeding throughout the follow-up as well as the trends in the number of infants attending each of the visits.

### 3.1. Psychomotor Development According to the Type of Breastfeeding at the Time of the Survey

At 4 months (Table 2), statistically significant differences were found for the milestone “recognizes bottle”, being 98.4% in the non-BF group compared to 91% in the BF group (*p* < 0.001); differences were also obtained for the milestone “transitions to sitting” and “forearm support”, being reached in the BF group with a higher percentage of children (*p* 0.046 and 0.041, respectively). At the 6-month visit (Table 3), no statistically significant differences were observed. At the one-year visit (Table 4) no differences were observed in any of the milestones, although for the milestone “understands a prohibition”, a difference of 95.9% was observed in the BF group compared to 93.6% in the non-BF group. For “points with the index finger”, a rate of 88.5% was observed in the BF group compared to 86.1% in the non-BF group.

At 18 months (Table 5), statistically significant differences (*p* 0.025) were observed for the milestone”feeds dolls”, with 90.2% in the BF group compared to 85.4% in the non-BF group. At 24 months (Table 6), statistically significant differences were observed for the milestone “descends stairs”, 99.2% in the BF group compared to 96.6% in the non-BF group.

### 3.2. Motor Development According to Type of Breastfeeding at 6 Months and Psychomotor Development at 12, 18 and 24 Months

When comparing the type of breastfeeding at 6 months and psychomotor development at 12 months (Table 7), no statistically significant differences were observed. At 18 months (Table 8), for the milestone “uses ‘no’”, 81.4% of the non-BF-at-6-months group exceeded this milestone compared to 85.8% of the BF-at-6-months group. At 24 months (Table 9), statistically significant differences were observed for the milestone “scribbles spontaneously”, achieved by 99.7% of the BF-at-6-months group compared to 97.9% of the non-BF group (*p* 0.007); for the milestone “descends stairs”, a statistically significant difference was also observed (*p* 0.002) in favour of the BF group (98.3%) compared to the non-BF group (95%).

### 3.3. Duration of Breastfeeding and Psychomotor Milestones at 12, 18 and 24 Months of Age

At 12 months (Table 10), a statistically significant difference was observed for the milestone “nonspecifically says ‘mom’ or ‘dad’”, with a mean duration of BF of 5.5 months for those who reached the milestone compared to 3.6 months for those who did not (*p* 0.02).

At 18 months (Table 11), statistically significant differences were found for the following milestones: “feeds dolls”(10.2 months of BF in the group that did reach it vs. 8.2 months in the group that did not (*p* 0.006)), and using “no” (10.2 months vs. 8.8 months (*p* 0.043).

At 24 months (Table 12), higher mean BF time was observed in the groups that did reach the following milestones: “eats with a spoon” (10.1 months vs. 4.7 months (*p* 0.007)) and “descends stairs” (10.2 months vs. 5.6 months (*p* 0.001)).

### 3.4. Duration of Exclusive Breastfeeding (EBF) and Psychomotor Milestones at 12, 18 and 24 Months

We again compared the EBF means of the groups that did reach the milestone vs. those that did not. At 12 months (Table 13), a statistically significant difference was observed for the milestone “nonspecifically says ‘mom’ or ‘dad’”, with a mean EBF of 5.5 months in those who reached it vs. 3.6 months in those who did not. At 18 months (Table 14), no statistically significant differences were found, but at 24 months (Table 15), higher mean EBFs were observed in the groups that did reach the following milestones: “eats with a spoon” (5.6 months vs. 2.4 months); “scribbles spontaneously” (5.6 months vs. 1.8 months); “makes towers with two cubes” (5.6 months vs. 0.8 months); and “descends stairs” (5.7 months vs. 4 months). Except for the 24-month milestones “takes off his pants” and “obeys orders with gestures”, the mean duration of EBF is higher in the ‘Yes’ group.

## 4. Discussion

In our study of a healthy population, there are no major findings on the relationship between BF and psychomotor development in the first two years of life. It is possible that the observed benefits in psychomotor development associated with BF are also related to other factors such as the social and economic context of each group; we list below a number of studies that reflect this. Colen and Ramey, in 2014 [15], conducted an initial study in which they showed that children aged 4 to 14 years who were fed BF obtained better results in terms of psychomotor development, but when they restricted their analysis to siblings within the same family, a clearer relationship was not observed, suggesting that the relationship could be influenced by social and economic status. Der et al. also found that, although they initially observed significant differences in cognitive and motor development in BF-fed children, when adjusting for maternal education level, family environment and other confounding factors, the differences are not as significant [11]. However, other studies, after adjusting for multiple confounding factors, do find a positive effect on the development of children with BF for 4 months or more [16].

In our study, the most striking differences accumulate at 24 months of age, and specifically, in the areas of manipulation, so it may be that longer-term follow-up is needed for these differences to be statistically significant. Boucher et al., in their prospective multicentre study in Spain, show that BF is independently associated with a small improvement in global cognitive function and fewer autistic traits at 4 years [8]; Julvez et al. observed that in two Spanish cohorts followed from 2 to 4 years of age, longer duration of BF was associated with fewer symptoms of attention deficit hyperactivity disorder [17]. Other studies find effects of duration of BF on the development of language and intelligence, but later in life (at 3 and 7 years), as in the case of Belfort et al. in their study of 1312 children [18]. However, Leventakou et al. conducted a study in 540 children, observing after adjustment for confounding factors that longer duration of BF was associated with higher cognitive, language and motor development scores at 18 months of age [19].

McGowan et al. reviewed all the literature published from 2013 to 2023 to examine the associations between BF and cognition, executive function and behaviour, showing that breastfeeding has a positive effect on IQ in later childhood and protects against behavioural disorders by achieving higher executive function [20], stages that would be beyond the scope of our study. In their study of 11,148 children in the United Kingdom followed up to the age of 14 years, Speyer et al. reflected on the relationship of the duration of BF with better socioemotional behavioural development of children in later childhood [21]. Fitzsimons E et al. show that, if the study population are families with low economic status, a relationship between breastfeeding and better cognitive development up to 7 years of age is observed [22].

Our study, due to the inclusion criteria, is a mostly healthy population in which the benefits of breastfeeding are not as striking in the early stages of life as they might be in populations with prenatal and/or neonatal risk factors. By not including patients requiring admission to neonatal units, premature infants, low birth weight, etc., a population clearly benefiting from breastfeeding is lost [23,24]. These inclusion criteria may be a limitation of our study as the beneficial effects of BF on psychomotor development are less visible; another limitation of our study is that only breastfeeding is taken into account and not the type of feeding from 6 months onwards, nor other factors that may or may not favour DPM (family, number of brothers, etc.). A study that includes more subjects and follows them for a longer period of time would probably be needed to make the benefits of breastfeeding more visible.

## 5. Conclusions

In our sample, psychomotor development in the first two years of life did not show significant differences in relation to feeding at 6 months of life and the duration of exclusive or non-exclusive BF. The fact that at 24 months of life, some further differences are observed may be because the effects on psychomotor development are more visible in the long term.

We believe that, as the literature indicates, many other social and economic factors influence psychomotor development in this period and that breastfeeding is not a determining factor in isolation.

## Figures and Tables

**Table 1:** Trends in breastfeeding reported at each visit.

	Visit 1 (15 Days)	Visit 2 (1 Month)	Visit 3 (2 Months)	Visit 4 (4 Months)	Visit 5 (6 Months)	Visit 6 (12 Months)	Visit 7 (18 Months)	Visit 8 (24 Months)
Number of cases	1946	1946	1807	1758	1681	1496	1250	1076
Type of feeding at each visit:								
EBF	66.4%	62.3%	60.2%	52.7%	35.2%	-	-	-
BF	22.7%	22.6%	19.1%	19%	26.5%	40.1%	30.2%	22.3%
Formula feeding (FF)	10.9%	15%	20.8%	28.3%	38.3%	59.9%	69.8%	77.7%

**Table 2:** Psychomotor milestones at 4-month visit (Visit 4) and feeding at that time.

	All Patients	Formula Feeding (FF)	Any Type of Breastfeeding	*p* Value
Reacts to voice	1756 (99.9%)	497 (100%)	1259 (99.8%)	1 **
Distinguishes his/her mother	1745 (99.7%)	494 (99.8%)	1251 (99.6%)	1 **
Recognizes bottle	1083 (94.2%)	*483 (98.4%)*	*600 (91.0%)*	<0.001 *
Looks at his/her hands	1698 (97.3%)	480 (97.2%)	1218 (97.4%)	0.820 *
Vertical persecution	1715 (98.6%)	488 (99%)	1227 (98.5%)	0.411 *
Attends to conversation	1709 (98.2%)	483 (98.4%)	1226 (98.1%)	0.685 *
Laughs out loud	1602 (92%)	448 (90.7%)	1154 (92.5%)	0.218 *
Joins hands	1655 (95.1%)	465 (94.3%)	1190 (95.4%)	0.371 *
Cephalic straightening	1736 (9959	491 (99%)	1245 (99.0%)	1 **
Transitions to sitting	1045 (61.9%)	*278 (58.2%)*	*767 (63.4%)*	0.046 *
Forearm support	1525 (87.5%)	*416 (84.9%)*	*1109 (88.5%)*	0.041 *

* Chi-square test. ** Fisher’s exact test. Statistically significant values are highlighted in italics.

**Table 3:** Psychomotor milestones at 6 months (Visit 5) and feeding at that time.

	All Patients	Formula Feeding (FF)	Any Type of Breastfeeding	*p* Value
Looks at his/her hands	1666 (99.3%)	634 (98.9%)	1032 (99.5%)	0.231 **
Vertical optical pursuit	1671 (99.6%)	639 (99.7%)	1032 (99.5%)	0.715 **
Horizontal optical pursuit	1673 (99.7%)	640 (99.8%)	1033 (99.6%)	0.655 **
Searches for fallen object	1541 (94%)	598 (94.5%)	943 (93.7%)	0.542 *
Eats cookie or other food	933 (77.3%)	372 (79.5%)	561 (75.9%)	0.149 *
Laughs out loud	1645 (98.1%)	632 (98.6%)	1013 (97.8%)	0.235 *
Babbles	1640 (97.8%)	630 (98.3%)	1010 (97.5%)	0.282 *
Directs the hand to object	1659 (99.1%)	636 (99.4%)	1023 (98.9%)	0.355 *
Changes object in hand	1483 (90.3%)	571 (91.2%)	912 (89.8%)	0.334 *
Removes scarf from face	1492 (91.6%)	580 (92.4%)	912 (91.2%)	0.412 *
Supports arms	1616 (96.6%)	613 (95.8%)	1003 (97.1%)	0.150 *
Parachute reflex	1230 (78%)	472 (77.4%)	758 (78.5%)	0.610 *

* Chi-square test. ** Fisher’s exact test.

**Table 4:** Psychomotor milestones at 12 months (Visit 6) and feeding at that time.

	All Patients	Formula Feeding (FF)	Any Type of Breastfeeding	*p* Value
Searches for fallen object	1490 (99.8%)	892 (99.7%)	598 (100%)	0.279 **
Plays hide and seek	1374 (94.1%)	817 (93.8%)	557 (94.6%)	0.542 *
Looks for a fallen object	1453 (98.4%)	865 (98.1%)	588 (99%)	0.163 *
Imitates simple gestures	1472 (98.5%)	882 (98.5%)	590 (98.5%)	0.937 *
Collaborates when dressed	1296 (88.5%)	777 (88.8%)	519 (88%)	0.624 *
Babbles	1490 (99.7%)	891 (99.6%)	599 (99.8%)	0.654 **
Nonspecifically says “mom” or “dad”	1443 (96.9%)	861 (96.7%)	582 (97.1%)	0.646 *
Understands a prohibition	1378 (94.5%)	815 (93.6%)	563 (95.9%)	0.054 *
Recognizes his name	1477 (99.1%)	883 (98.9%)	594 (99.5%)	0.209 *
Understands the meaning of some words	1425 (97.6%)	849 (97.5%)	576 (97.8%)	0.696 *
Obeys orders with gestures	1300 (91.2%)	767 (90.7%)	533 (92.1%)	0.361 *
Changes handheld objects	1490 (100%)	892 (100%)	598 (100%)	-
Removes scarf from face	1487 (99.6%)	889 (99.4%)	598 (99.8%)	0.411 **
Performs inferior pincer grasp	1426 (98.3%)	853 (98.3%)	573 (98.5%)	0.790 *
Performs superior pincer grasp	1430 (97.5%)	855 (97.6%)	575 (97.5%)	0.860 *
Points with index finger	1266 (87.1%)	748 (86.1%)	518 (88.5%)	0.168 *
Performs lateral parachute	1422 (98.5%)	847 (98.4%)	575 (98.8%)	0.510 *
Independent sitting	1480 (99.3%)	885 (99%)	595 (99.7%)	0.217 **
Stands with support	1444 (96.7%)	864 (96.6%)	580 (96.8%)	0.845 *
Sits without support	1456 (97.7%)	874 (97.8%)	582 (97.7%)	0.887 *

* Chi-square test. ** Fisher’s exact test.

**Table 5:** Psychomotor milestones at 18 months (Visit 7) and feeding at that time.

	All Patients	Formula Feeding (FF)	Any Type of Breastfeeding	*p* Value
Imitates gestures	1246 (99.8%)	870 (99.9%)	376 (99.5%)	0.219 **
Cooperates with dressing	1233 (99%)	863 (99.2%)	370 (98.7%)	0.361 **
Takes glass to mouth	1235 (99.3%)	859 (99.1%)	376 (99.7%)	0.291 **
Imitates household tasks	1202 (97%)	836 (96.9%)	366 (97.3%)	0.656 *
Feeds dolls	996 (86.8%)	682 (85.4%)	314 (90.2%)	0.025 *
Understands prohibitions	1230 (99.2%)	858 (99.1%)	372 (99.5%)	0.482 *
Understands the meaning of words	1242 (99.6%)	866 (99.5%)	376 (99.7%)	1 **
Obeys orders with gestures	1213 (98.6%)	845 (98.3%)	368 (99.5%)	0.097 *
Says “mom” or “dad” appropriately	1184 (95.2%)	828 (95.4%)	356 (94.7%)	0.591 *
Uses “no”	1040 (84.2%)	715 (83.1%)	325 (86.7%)	0.118 *
Points out parts of body	1134 (92.4%)	795 (92.4%)	339 (92.4%)	0.966 *
Performs superior pincer grasp	1233 (99%)	860 (99%)	378 (98.9%)	0.968 **
Points with index finger	1234 (99%)	861 (99.1%)	373 (98.7%)	0.549 **
Scribbles	1195 (97%)	832 (97%)	363 (97.1%)	0.933 *
Turns pages of a book	1187 (96.7%)	825 (96.5%)	362 (97.1%)	0.616 *
Makes towers with four cubes	1165 (96.6%)	815 (97.1%)	350 (95.4%)	0.118 *
Places a cap on a pen	1035 (91%)	717 (90.8%)	318 (91.6%)	0.631 *
Builds a tower with four blocks	882 (78.2%)	627 (79.3%)	255 (75.7%)	0.182*
Sits independently	1247 (99.8%)	871 (99.9%)	376 (99.5%)	0.219 **
Takes five steps alone	1229 (98.4%)	860 (98.7%)	369 (97.6%)	0.148 *
Walks independently	1225 (98.1%)	858 (98.5%)	367 (97.1%)	0.094 *
Stands independently	1240 (99.3%)	865 (99.3%)	375 (99.2%)	1 **
Runs	1167 (94%)	816 (94.3%)	351 (93.4%)	0.501 *
Walks backwards	958 (84.6%)	671 (84.6%)	287 (84.4%)	0.931 *

* Chi-square test. ** Fisher’s exact test. Statistically significant values are highlighted in italics.

**Table 6:** Psychomotor milestones at 24 months (Visit 8) and feeding at that time.

	All Patients	Formula Feeding (FF)	Any Type of Breastfeeding	*p* Value
Imitates household tasks	1058 (98.9%)	820 (98.8%)	238 (99.2%)	1 **
Eats with a spoon	1061 (98.8%)	821 (98.4%)	240 (100%)	0.085 **
Helps to collect toys	1039 (97.4%)	811 (97.7%)	228 (96.2%)	0.200 *
Feeds dolls	985 (95.1%)	760 (94.4%)	225 (97.4%)	0.064 *
Takes off pants	884 (84.4%)	689 (84.9%)	195 (82.6%)	0.408 *
Obeys orders with gestures	1043 (98.4%)	814 (98.5%)	229 (97.9%)	0.554 *
Uses “no”	1050 (97.8%)	813 (97.5%)	237 (98.8%)	0.242 *
Points out parts of the body	1053 (98.2%)	816 (98.1%)	237 (98.8%)	0.591 *
Names a drawn object	968 (91.4%)	749 (91%)	219 (92.8%)	0.388 *
Follows two-step commands	1016 (97%)	789 (97%)	227 (97%)	0.975 *
Combines two words	930 (87%)	716 (86.3%)	214 (89.5%)	0.185 *
Uses pronouns	787 (75.7%)	612 (75.5%)	175 (76.8%)	0.688 *
Names five images	909 (86.7%)	705 (86.5%)	204 (87.6%)	0.677 *
Scribbles spontaneously	1060 (99.1%)	823 (99%)	237 (99.2%)	0.859 *
Turns pages one by one	1055 (99%)	819 (99%)	236 (98.7%)	0.717 **
Makes towers with two cubes	1059 (99.2%)	823 (99%)	236 (99.6%)	0.693 **
Places a cap on a pen	998 (96.9%)	772 (96.6%)	226 (97.8%)	0.349 *
Makes towers with four cubes	981 (95.2%)	765 (95.5%)	216 (93.9%)	0.322 *
Runs	1061 (98.8%)	822 (98.6%)	239 (99.6%)	0.318 **
Walks backwards	968 (94.8%)	745 (94.4%)	223 (96.1%)	0.306 *
Descends stairs	1037 (97.2%)	*800 (96.6%)*	*237 (99.2%)*	0.036 *
Shoots at a ball	1043 (97.5%)	814 (98%)	229 (95.8%)	0.063 *
Jumps forward	926 888.6%)	718 (88.4%)	208 (89.3%)	0.720 *

* Chi-square test. ** Fisher’s exact test. Statistically significant values are highlighted in italics.

**Table 7:** Psychomotor milestones at 12 months (Visit 6) and BF at 6 months.

	All Patients	No Breastfeeding	Any Type of Breastfeeding	*p* Value
Searches for fallen object	1432 (99.9%)	535 (100%)	897 (99.8%)	0.532 **
Eats a cookie	1413 (99.1%)	523 (98.5%)	890 (99.4%)	0.085 **
Plays hide and seek	1316 (93.9%)	483 (93.1%)	833 (94.4%)	0.296 *
Looks for a fallen object	1395 (98.5%)	520 (98.7%)	875(98.4%)	0.711 *
Imitates simple gestures	1415 (98.7%)	527 (98.5%)	888 (98.8%)	0.663 *
Collaborates when dressed	1250 (88.7%)	459 (87.3%)	791 (89.6%)	0.183 *
Babbles	1433 (99.9%)	535 (100%)	898 (99.8%)	0.532 **
Nonspecifically says “mom” or “dad”	1387 (97.1%)	514 (96.6%)	873 (97.3%)	0.444 *
Understands a prohibition	1323 (94.5%)	491 (94.4%)	832 (94.559	0.923 *
Recognizes his/her name	1419 (99.2%)	530 (99.4%)	889 (99.1%)	0.756 **
Understands the meaning of some words	1369 (97.8%)	511 (97.7%)	858 (97.8%)	0.875 *
Obeys orders with gestures	1250 (91.2%)	452 (90.4%)	798 (91.7%)	0.404 *
Changes handheld objects	1432 (100%)	536 (100%)	896 (100%)	-
Removes scarf from face	1429 (99.7%)	535 (99.8%)	894 (99.7%)	1 **
Performs inferior pincer grasp	1372 (98.5%)	510 (98.8%)	862 (98.3%)	0.418 *
Performs superior pincer grasp	1378 (97.7%)	513 (98.1%)	865 (97.5%)	0.489 *
Points with index finger	1214 (87%)	456 (97.7%)	758 (86.8%)	0.568 *
Performs lateral parachute	1367 (98.6%)	506 (99.0%)	861 (98.4%)	0.337 *
Independent sitting	1422 (99.4%)	531 (99.4%)	891 (99.3%)	1 **
Stands with support	1387 (96.8%)	514 (96.3%)	873 (97.1%)	0.376 *
Sits without support	1399 (97.8%)	525 (98.1%)	874 (97.5%)	0.468 *

* Chi-square test. ** Fisher’s exact test.

**Table 8:** Psychomotor milestones at 18 months (Visit 7) and BF at 6 months.

	All Patients	No Breastfeeding	Any Type of Breastfeeding	*p* Value
Imitates gestures	1198 (99.8%)	434 (99.6%)	764 (99.7%)	1 **
Cooperates with dressing	1185 (99%)	431 (99.3%)	754 (98.8%)	0.553 **
Takes glass to mouth	1187 (99.2%)	428 (99.1%)	759 (99.3%)	0.730 **
Imitates household tasks	1155 (97.0%)	414 (96.7%)	741 (97.1%)	0.708 *
Feeds dolls	956 (86.6%)	342 (85.1%)	614 (87.5%)	0.262 *
Understands prohibitions	1183 (99.2%)	430 (99.5%)	753 (98.9%)	0.344 **
Understands the meaning of words	1194 (99.6%)	433 (99.8%)	761 (99.5%)	0.659 **
Obeys orders with gestures	1166 (98.6%)	422 (98.8%)	744 (98.5%)	0.683 *
Says “mom” or “dad” appropriately	1136 (95.0%)	413 (95.4%)	723 (94.8%)	0.635 *
Uses “no”	1001 (84.2%)	*351 (81.4%)*	*650 (85.8%)*	0.050 *
Points out parts of his body	1091 (92.4%)	393 (91.4%)	698 (92.9%)	0.335 *
Performs superior pincer grasp	1185 (98.9%)	431 (99.1%)	754 (98.8%)	0.779 **
Points with index finger	1186 (98.9%)	429 (99.1%)	757 (98.8%)	0.779 **
Scribbles	1150 (97.1%)	414 (96.3%)	736 (97.6%)	0.186 *
Turns pages of a book	1141 (96.7%)	409 (96.5%)	732 (96.8%)	0.738 *
Makes towers with four cubes	1121 (96.6%)	398 (96.1%)	723 (96.8%)	0.560 *
Places a cap on a pen	995 (90.9%)	349 (89.7%)	646 (91.5%)	0.327 *
Builds a tower with four blocks	849 (78.0%)	303 (78.3%)	546 (77.9%)	0.877 *
Sits independently	1199 (99.8%)	435 (99.8%)	764 (99.7%)	1 **
Takes five steps alone	1181 (98.3%)	427 (98.2%)	754 (98.4%)	0.723 *
Walks independently	1177 (98.0%)	426 (97.9%)	751 (98.0%)	0.895 *
Stands independently	1192 (99.3%)	429 (98.6%)	763 (99.6%)	0.079 **
Runs	1122 (94.0%)	407 (94.0%)	715 (94.0%)	0.978 *
Walks backwards	923 (84.4%)	331 (83.4%)	592 (84.9%)	0.494 *

* Chi-square test. ** Fisher’s exact test. Statistically significant values are highlighted in italics.

**Table 9:** Psychomotor milestones at 24 months (Visit 8) and BF at 6 months.

	All Patients	No Breastfeeding	Any Type of Breastfeeding	*p* Value
Imitates household tasks	1017 (98.8%)	374 (98.2%)	643 (99.2%)	0.140 *
Eats with a spoon	1021 (98.8%)	376 (97.9%)	645 (99.4%)	0.066 **
Helps to collect toys	999 (97.4%)	368 (96.6%)	631 (97.8%)	0.230 *
Feeds dolls	947 (94.9%)	349 (94.1%)	598 (95.4%)	0.366 *
Takes off pants	849 (84.3%)	324 (86.2%)	525 (83.2%)	0.210 *
Obeys orders with gestures	1002 (98.3%)	375 (98.2%)	627 (98.4%)	0.751 *
Uses “no”	1010 (97.7%)	373 (97.1%)	637 (98.0%)	0.372 *
Points out parts of the body	1013 (98.3%)	378 (98.4%)	635 (98.1%)	0.729 *
Names a drawn object	930 (91.3%)	339 (90.2%)	591 (91.9%)	0.339 *
Follows two-step commands	976 (97%)	362 (97.3%)	614 (96.8%)	0.675 *
Combines two words	893 (86.9%)	328 (85.6%)	565 (87.6%)	0.369 *
Uses pronouns	754 (75.8%)	272 (73.3%)	482 (76.9%)	0.206 *
Names five images	872 (86.5%)	322 (85.9%)	550 (86.9%)	0.646 *
Scribbles spontaneously	1019 (99%)	*376 (97.9%)*	*643 (99.7%)*	0.007 **
Turns pages one by one	1015 (98.9%)	374 (98.2%)	641 (99.4%)	0.112 **
Makes towers with two cubes	1018 (99.1%)	375 (98.4%)	643 (99.5%)	0.085 **
Places a cap on a pen	958 (96.8%)	353 (96.7%)	605 (96.8%)	0.940 *
Makes towers with four cubes	943 (95%)	346 (93.8%)	597 (95.7%)	0.184 *
Runs	1020 (98.7%)	375 (97.9%)	645 (99.2%)	0.083 **
Walks backwards	932 (94.6%)	336 (92.8%)	596 (95.7%)	0.056 *
Descends stairs	996 (97.1%)	*361 (95.0%)*	*635 (98.3%)*	0.002 *
Shoots at a ball	1002 (97.4%)	368 (96.8%)	634 (97.7%)	0.412 *
Jumps forward	888 (88.3%)	331 (88.5%)	557 (88.1%)	0.860 *

* Chi-square test. ** Fisher’s exact test. Statistically significant values are highlighted in italics.

**Table 10:** Comparison of means of duration of BF (any type of BF: exclusive and non-exclusive) in months for those who reach or do not reach the psychomotor milestones at 12 months (Visit 6).

	Yes	No	*p* Value
*Searches for fallen object*	*9.4*	*5.6*	*0.020*
Plays hide and seek	9.5	9.0	0.631
Looks for a fallen object	9.4	7.6	0.267
Imitates simple gestures	9.4	9.5	0.918
Collaborates when dressed	9.4	9.3	0.918
Babbles	9.4	6.9	0.510
Nonspecifically says “mom” or “dad”	9.4	8.4	0.419
Understands a prohibition	9.5	7.9	0.080
Recognizes his/her name	9.4	8.5	0.722
Understands the meaning of some words	9.4	7.7	0.152
Obeys orders with gestures	9.5	8.9	0.426
Changes handheld objects	9.4	-	-
Removes scarf from face	9.4	5.7	0.104
Performs inferior pincer grasp	9.4	9.1	0.851
Performs superior pincer grasp	9.4	8.6	0.602
Points with index finger	9.5	8.6	0.149
Performs lateral parachute	9.4	8.4	0.580
Independent sitting	9.4	6.4	0.084
Stands with support	9.4	8.8	0.636
Sits without support	9.4	8.9	0.736

Student’s *t*-test. Statistically significant values are highlighted in italics.

**Table 11:** Comparison of means of duration of BF (any type of BF: exclusive and non-exclusive) in months for those who reach or do not reach the psychomotor milestones at 18 months (Visit 7).

	Yes	No	*p* Value
Imitates gestures	10.0	10.3	0.956
Cooperates with dressing	9.9	11.5	0.535
Takes glass to mouth	10.0	6.4	0.228
Imitates household tasks	10.0	9.5	0.715
Feeds dolls	*10.2*	*8.2*	*0.006*
Understands prohibitions	10.0	8.7	0.655
Understands the meaning of words	10.0	7.6	0.238
Obeys orders with gestures	10.0	7.3	0.126
Says “mom” or “dad” appropriately	10.0	10.0	0.971
*Uses “no”*	*10.2*	*8.8*	*0.043*
Points out parts of his body	10.0	9.9	0.922
Performs superior pincer grasp	10.0	10.9	0.707
Points with index finger	10.0	10.9	0.700
Scribbles	10.0	8.5	0.286
Turns pages of a book	10.0	10.0	0.976
Makes towers with four cubes	10.0	10.9	0.504
Places a cap on a pen	10.1	9.6	0.591
Builds a tower with four blocks	9.8	10.5	0.244
Sits independently	10.0	14.6	0.361
Takes five steps alone	9.9	12.6	0.306
Walks independently	9.9	12.3	0.201
Stands independently	10.0	7.1	0.321
Runs	9.9	10.7	0.447
Walks backwards	10.0	9.5	0.431

Student’s *t*-test. Statistically significant values are highlighted in italics.

**Table 12:** Comparison of means of duration of BF (any type of BF: exclusive and non-exclusive) in months for those who reach or do not reach the psychomotor milestones at 24 months (Visit 8).

	Yes	No	*p* Value
Imitates household tasks	10.1	8.8	0.629
*Eats with a spoon*	*10.1*	*4.7*	*0.007*
Helps to collect toys	10.0	10.8	0.711
Feeds dolls	10.1	8.7	0.281
Takes off pants	9.9	10.9	0.212
Obeys orders with gestures	9.9	11.2	0.557
Uses “no”	10.0	10.1	0.980
Points out parts of the body	10.0	9.6	0.852
Names a drawn object	10.1	9.3	0.425
Follows two-step commands	10.0	11.6	0.341
Combines two words	10.1	9.1	0.221
Uses pronouns	10.1	9.4	0.290
Names five images	10.0	9.8	0.782
Scribbles spontaneously	10.0	6.6	0.242
Turns pages one by one	10.0	8.4	0.562
Makes towers with two cubes	10.0	5.7	0.161
Places a cap on a pen	10.1	8.8	0.442
Makes towers with four cubes	10.0	9.8	0.873
Runs	10.1	5.8	0.064
Walks backwards	10.2	8.8	0.275
*Descends stairs*	*10.2*	*5.6*	*0.001*
Shoots at a ball	10.0	11.8	0.423
Jumps forward	10.0	10.4	0.623

Student’s *t*-test. Statistically significant values are highlighted in italics.

**Table 13:** Comparison of means of duration of EBF in months for those who reach or do not reach psychomotor milestones at 12 months (Visit 6).

	Yes	No	*p* Value
Searches for fallen object	5.5	2.1	0.425
Plays hide and seek	5.6	4.2	0.092
Looks for a fallen object	5.5	4.1	0.465
Imitates simple gestures	5.4	5.4	0.930
Collaborates when dressed	5.5	5.0	0.430
Babbles	5.5	4.4	0.970
*Nonspecifically says “mom” or “dad”*	*5.5*	*3.6*	*0.029*
Understands a prohibition	5.6	4.1	0.431
Recognizes his/her name	5.4	6.6	0.594
Understands the meaning of some words	5.5	3.9	0.417
Obeys orders with gestures	5.6	5.3	0.923
Changes handheld objects	5.5	-	-
Removes scarf from face	5.4	3.3	0.628
Performs inferior pincer grasp	5.5	4.9	0.865
Performs superior pincer grasp	5.5	5.6	0.728
Points with index finger	5.5	5.2	0.742
Performs lateral parachute	5.5	5.6	0.462
Independent sitting	5.5	3.3	0.299
Stands with support	5.5	4.6	0.669
Sits without support	5.4	5.1	0.946

Mann–Whitney U test. Statistically significant values are highlighted in italics.

**Table 14:** Comparison of means of duration of EBF in months for those who reach or do not reach psychomotor milestones at 18 months (Visit 7).

	Yes	No	*p* Value
Imitates gestures	5.6	2.0	0.236
Cooperates with dressing	5.6	7.3	0.436
Takes glass to mouth	5.6	5.9	0.971
Imitates household tasks	5.7	4.4	0.193
Feeds dolls	5.7	4.7	0.080
Understands prohibitions	5.6	5.2	0.988
Understands the meaning of words	5.6	4.8	0.878
Obeys orders with gestures	5.6	4.8	0.608
Says “mom” or “dad” appropriately	5.7	4.3	0.255
Uses “no”	5.7	5.0	0.223
Points out parts of body	5.6	5.7	0.795
Performs superior pincer grasp	5.6	5.3	0.587
Points with index finger	5.6	6.1	0.548
Scribbles	5.7	4.1	0.261
Turns pages of a book	5.7	4.6	0.619
Makes towers with four cubes	5.6	6.3	0.736
Places a cap on a pen	5.8	5.3	0.309
Builds a tower with four blocks	5.4	6.3	0.693
Sits independently	5.6	8.6	0.307
Takes five steps alone	5.6	4.5	0.367
Walks independently	5.6	4.3	0.367
Stands independently	5.6	3.3	0.149
Runs	5.6	5.2	0.323
Walks backwards	5.7	5.2	0.268

Mann–Whitney U test.

**Table 15:** Comparison of means of duration of EBF in months for those who reach or do not reach psychomotor milestones at 24 months (Visit 8).

	Yes	No	*p* Value
Imitates household tasks	5.6	3.6	0.222
*Eats with a spoon*	*5.6*	*2.4*	*0.014*
Helps to collect toys	5.6	4.7	0.094
Feeds dolls	5.7	4.4	0.183
Takes off pants	5.5	6.1	0.239
Obeys orders with gestures	5.5	5.6	0.972
Uses “no”	5.6	3.6	0.332
Points out parts of the body	5.6	5.3	0.997
Names a drawn object	5.7	4.0	0.051
Follows two-step commands	5.6	4.7	0.365
Combines two words	5.7	4.6	0.138
Uses pronouns	5.7	5.0	0.150
Names five images	5.6	5.1	0.451
*Scribbles spontaneously*	*5.6*	*1.8*	*0.021*
Turns pages one by one	5.6	3.9	0.242
*Makes towers with two cubes*	*5.6*	*0.8*	*0.004*
Places a cap on a pen	5.7	4.3	0.496
Makes towers with four cubes	5.6	4.0	0.089
Runs	5.6	2.0	0.053
Walks backwards	5.7	4.3	0.102
*Descends stairs*	*5.7*	*3.3*	*0.010*
Shoots at a ball	5.6	6.0	0.655
Jumps forward	5.7	4.9	0.299

Mann–Whitney U test. Statistically significant values are highlighted in italics.

## Data Availability

The original contributions presented in this study are included in the article. Further inquiries can be directed to the corresponding author.

## Abbreviations

The following abbreviations are used in this manuscript:

BF	Breastfeeding
EBF	Exclusive breastfeeding
FF	Formula feeding
